# A Few-Shot U-Net Deep Learning Model for COVID-19 Infected Area Segmentation in CT Images

**DOI:** 10.3390/s21062215

**Published:** 2021-03-22

**Authors:** Athanasios Voulodimos, Eftychios Protopapadakis, Iason Katsamenis, Anastasios Doulamis, Nikolaos Doulamis

**Affiliations:** 1 Department of Informatics and Computer Engineering, University of West Attica, 12243 Athens, Greece; eprotopapadakis@uniwa.gr; 2 School of Rural and Surveying Engineering, National Technical University of Athens, 15780 Athens, Greece; iasonkatsamenis@mail.ntua.gr (I.K.); adoulam@cs.ntua.gr (A.D.); ndoulam@cs.ntua.gr (N.D.)

**Keywords:** deep learning, few-shot learning, semantic segmentation, CT images, COVID-19

## Abstract

Recent studies indicate that detecting radiographic patterns on CT chest scans can yield high sensitivity and specificity for COVID-19 identification. In this paper, we scrutinize the effectiveness of deep learning models for semantic segmentation of pneumonia-infected area segmentation in CT images for the detection of COVID-19. Traditional methods for CT scan segmentation exploit a supervised learning paradigm, so they (a) require large volumes of data for their training, and (b) assume fixed (static) network weights once the training procedure has been completed. Recently, to overcome these difficulties, few-shot learning (FSL) has been introduced as a general concept of network model training using a very small amount of samples. In this paper, we explore the efficacy of few-shot learning in U-Net architectures, allowing for a dynamic fine-tuning of the network weights as new few samples are being fed into the U-Net. Experimental results indicate improvement in the segmentation accuracy of identifying COVID-19 infected regions. In particular, using 4-fold cross-validation results of the different classifiers, we observed an improvement of 5.388 ± 3.046% for all test data regarding the IoU metric and a similar increment of 5.394 ± 3.015% for the F1 score. Moreover, the statistical significance of the improvement obtained using our proposed few-shot U-Net architecture compared with the traditional U-Net model was confirmed by applying the Kruskal-Wallis test (*p*-value = 0.026).

## 1. Introduction

The novel coronavirus 2019-nCoV, now SARS-CoV-2, was first transmitted to humans in December 2019, resulting in a pandemic outbreak in the following months. The disease, known as COVID-19 [1], has already caused significant short-term and long-term societal and economic impacts [2], resulting in more than 2,600,000 deaths up to 8 March 2021 [3]. Further insight into the findings so far indicates that it affects multiple organs, including the heart and blood vessels, kidneys, intestines, and brain. The virus enters cells by binding to surface angiotensin-converting enzyme 2 (ACE2) receptors, which can be found on alveoli, i.e., tiny air sacs in the lungs, making them ground zero for infection [4].

Medical imaging, such as Computed Tomography (CT) scanning, along with Artificial Intelligence (AI) technologies, is a promising and efficient alternative tool for the detection and control of COVID-19. Usually, disease is detected using a RT-PCR test (Reverse Transcription-Polymerase Chain Reaction); however, it requires (a) 4–6 h to be completed; (b) specialized medical equipment, staff, and resources; and (c) it suffers from sample preparation issues [5]. An alternative approach used in clinical settings is using CT scans to observe the characteristic appearance indicators of COVID-19’s effect on the lungs [6]. However, the automatic examination of CT scans can be seen as an image analysis problem that can be solved using annotated data and machine-learning algorithms like deep learning [7].

Recently, many research works have been proposed for applying deep learning models for COVID-19 infected area segmentation. An interesting review in this research field is the work of [6]. Usually, Fully Convolutional Networks (FCNs) or U-shaped Convolutional Networks (U-Nets) are considered for providing a pixel-based segmentation of COVID-19 infected regions from CT scans. This is because FCNs and U-Nets have emerged as powerful segmentation tools, especially for performing an accurate pixel-based segmentation of medical data expressed either in 2D or 3D [8]. For this reason, these networks have also been considered as one of the first approaches for efficiently segmenting regions infected by COVID-19 from CT scans [9,10,11].

**Figure 1:**
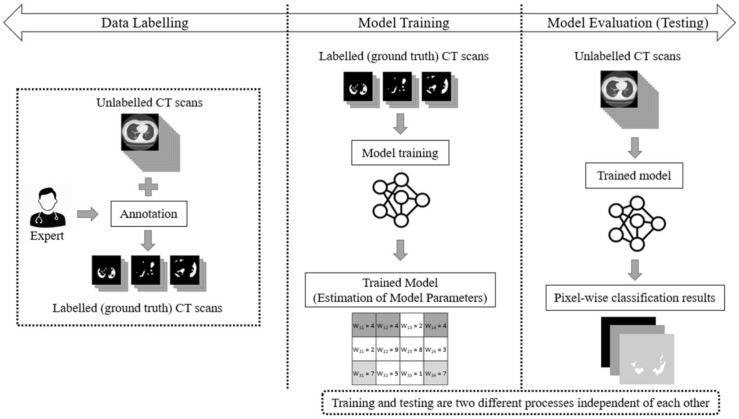
The supervised learning approach for the segmentation task of COVID-19 CT scans, consisting of three main phases: data labelling, model training, and model testing (evaluation).

The main drawback of the aforementioned supervised learning paradigms is that they handle COVID-19 segmentation as a static process. Usually, in supervised learning, three main phases are considered: data labelling, model training, and model testing (evaluation). In a supervised learning paradigm, training and testing are two independent processes. Initially, experts annotate a pool of unlabelled data (e.g., CT scans), thereby creating labelled (ground truth) datasets. Then, the model is trained and its parameters (weights) are estimated through the use of a learning algorithm. During the evaluation phase, the algorithm performs the CT scan segmentation task based on the learned model weights. However, once the network has been trained using ground-truth data labels, its parameters (weights) remain fixed (static). This implies that the performance of the deep model is finalized and cannot be improved once the network has been trained. The main phases of a traditional supervised learning paradigm are depicted in Figure 1.

Usually, supervised learning assumes large volumes of data in the training set in order to cover all potential variations. However, collecting large amounts of training data is an arduous task, especially in the medical imaging domain, since a manual annotation procedure is required. One way to address this difficulty is to apply few-shot learning (FSL): feeding a learning model with a very small amount of training data, contrary to the normal practice of using a large amount of data [12]. As is presented in [13], few-shot learning was implemented using several variants, which can be categorized into three main perspectives: data, model, and algorithm. The data-perspective algorithms often augment the training dataset by means of prior knowledge. The model perspective methods constrain the classification space using embedding (e.g., learning). Finally, the algorithm perspective approaches refine (fine-tune) existing network parameters by exploiting knowledge from few incoming samples. Thus, FSL refers to a general concept of algorithms/techniques (like the concept of supervised/unsupervised learning), not to a specific learning algorithm. Additionally, the application of FSL to different deep learning classifiers (networks) requires different formulation, modelling and configuration setups.

**Figure 2:**
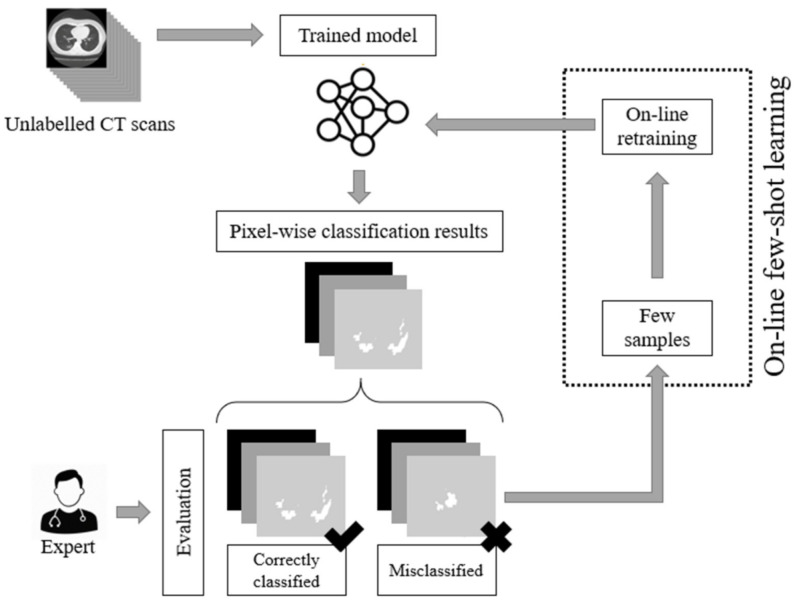
The proposed on-line few-shot learning paradigm. During the testing phase, experts are capable of evaluating the results of the network. This way, labelled samples are selected. These samples are used for the dynamic updating of the model parameters (weights) of the model, resulting in a dynamic (on-line) few-shot learning paradigm.

As we have previously stated, the current deep learning models used for COVID-19 infected area segmentation in CT scans are usually based on U-Net and FCN structures trained through a supervised learning paradigm. Therefore, no dynamic network weight readjustment is supported. In addition, there are issues with respect to the large number of data samples required for training. In this paper, we addressed this limitation by introducing an on-line few-shot learning paradigm where the network was dynamically trained, as few samples are being fed to the model. Our approach actually lies in the few-shot learning taxonomy since network weights are dynamically refined as few new data (samples) are feeding the model. This on-line few-shot learning technique is depicted in Figure 2. The proposed dynamic adjustment of the model weights is unique and novel since, to the best of our knowledge, no few-shot U-Net scheme (in the sense of dynamic learning during the testing phase) exists, let alone one for segmenting lung CT scans, especially regions infected by COVID-19.

To this end, a trained U-Net model was initially used to perform the segmentation of CT scans into regions infected or not infected by COVID-19. Then, during the testing phase, experts evaluated the network results, pointing out correctly classified or misclassified outputs. In the sequel, few samples were selected for further network training through an on-line few-shot learning process. As few new ground-truth data were inserted into the training set, the deep model learned to modify its behavior dynamically to trust the incoming knowledge with minimum modification of the previously gained one. The proposed few-shot U-Net model is capable of dynamically readjusting its parameters according to user feedback to further increase the performance of the segmentation, especially in cases where low performance is encountered. The Python code of the experiments and implementation, along with the trained deep learning models, are made available online: https://github.com/ikatsamenis/COVID19-few-shot-CT-segmentation (accessed date 20 March 2021).

## 2. Related Work

CT abnormalities related to COVID-19 patients are common, reported, and used by doctors in multiple studies [14,15,16]. There are two important outcomes from these studies: (a) there are clear patterns indicating viral infections, even at an early stage [15,16]; (b) CT abnormalities in diagnosing viral pneumonia can be available before a positive laboratory test in almost 70% of cases [16]. Hence, CT investigation appears to be a promising candidate for early detection of COVID-19 infection. There have also been several works that discuss COVID-19 detection in chest X-ray images (e.g., [7,17]) but here we focused on works using CT scans.

Research outcomes on COVID-19 confirmed cases indicated that CT abnormalities before the appearance of clinical symptoms may occur [18]. Asymptomatic patients typically have abnormal chest CTs, which is consistent with viral pneumonia. On the one hand, typical patterns may refer to unilateral, multifocal and peripherally based ground-glass opacities (GGO). On the other hand, interlobular septal thickening, thickening of the adjacent pleura, nodules, round cystic changes, bronchiectasis, pleural effusion, and lymphadenopathy were rarely observed in the asymptomatic group but appeared in symptomatic cases.

The adaptation of any visual detection approach should emphasize the identification of predominant patterns of lung abnormalities like GGOs, crazy-paving pattern, consolidation, and linear opacities. Yet, the appearance rates and the density varied greatly depending on the stage of the disease. A maximum manifestation was expected 9 days after the onset of initial symptoms [15].

Deep learning approaches over various types of images are common for identification, detection, or segmentation in medical imaging [19] and biomedical applications in general [20]. In this context, researchers have already started investigating several approaches to assist medical professionals with COVID-19 detection.

An initial approach was to classify multiple CT slices using a convolutional neural network variation [14]. The adopted methodology is able to identify a viral infection with a ROCAUC score of 0.95: a score of 1 indicates a perfect classifier. However, despite the high detection rates, the authors indicated that was extremely difficult to distinguish among different types of viral pneumonia based solely on CT analysis.

Convolutional Neural Network (CNN) variations for the distinction of coronavirus vs. non-coronavirus cases have been proposed by [21]. The specific approach allows for a distinction among COVID-19, other types of viral infections, and non-infection cases. Results indicate that there are adequate detection rates and a higher detection rate than RT-PCR testing. Towards this direction, CNN structures are combined with Long Short Term Memory (LSTM) networks to improve the classification accuracy of CNN networks [22] further. The work of [23] introduced a parallel partial decoder, called Inf-Net, which combines aggregation of high-level features to generate a global map. This is achieved through the use of convolutional hierarchies.

An alternative approach is the use of U-Net structures. U-Net is a convolutional neural network mainly designed for medical applications [24]. To this end, a multistage approach involving segmentation and classification between COVID-19 and other viral infection has been proposed in [25], allowing for advanced disease progression monitoring. In [6], there is a brief review description of the methods used for COVID-19 image segmentation, which is mainly focused on U-Net structures. At first, a segmentation approach (a U-Net) focuses on the lungs’ region by removing image portions that are not relevant for detection. Then, a pretrained Resnet-50 network is modified to handle the classification into COVID-19 or other cases [26]. In this context, a multi-objective adaptive convolutional neural network, called AdaResU-Net was proposed in [27] to consider an automatic adaptation to new datasets by performing a residual learning paradigm. A U-Net-based model, named U-Net++, was applied to high-resolution CT images for COVID-19 detection in [28]. Furthermore, in [29] a system for the detection of COVID-19 using 10 variants of CNNs in CT images is proposed, including AlexNet, VGG-16, VGG-19, SqueezeNet, GoogleNet, MobileNet-V2, ResNet-18, ResNet-50, ResNet-101, and Xception. ResNet-101 and Xception outperformed the remaining ones. AlexNet and Inception-V4 were also used for COVID-19 detection in CT scans in [30]. The framework presented in [31] used a CNN and an Artificial Neural Network Fuzzy Inference System (ANNFIS) to detect COVID-19, whereas a Stack Hybrid Classification (SHC) scheme based on ensemble learning is proposed in [32].

Moreover, an object-detection approach (denoting the areas of interest using bounding boxes) was also considered [33]. The detection of symptomatic lung areas was achieved by employing a VGG architecture variation [34]. The proposed approach can classify COVID-19 cases from community-acquired pneumonia (CAP) and non-pneumonia (NP). In [35], discrete wavelet transform and extended segmentation-based fractal texture analysis methods are used for feature extraction, followed by a genetic algorithm for feature selection and a Naïve Bayes classifier for COVID-19 diagnosis.

Focusing on segmentation, a type 2 fuzzy clustering system combined with a Super-pixel based Fuzzy Modified Flower Pollination Algorithm is proposed in [36] for COVID-19 CT image segmentation. Volumetric Medical Image segmentation networks, known as V-Nets [37], were also used as an alternative powerful toolkit for segmentation of COVID-19 images. In this context, the work of [38], used a V-Net to segment all the slices of a given MRI at once. Quantitative evaluation results indicate that automatic infection-region delineation can be feasible and effective. Moreover, deep learning techniques have been proposed for infection segmentation in lung CT images [39] to evaluate disease severity and quantify infection levels [40,41]. Table 1 provides a tabulated summary of machine learning techniques employed for COVID-19 detection and segmentation in CT images.

A major limitation of the aforementioned studies, especially the deep-learning-based ones, is that they required a large amount of ground-truth data for training. However, acquiring such large amounts of annotated data can be time-consuming, labor-intensive or, in many cases, impossible due to the very nature of the data. This fact significantly limits the applicability and utility of the approaches in real-world settings. This issue has started to be addressed in a very limited number of works (e.g., in [23]) where a semi-supervised learning scheme was employed that first segments infection regions to use them to drive the subsequent multi-class segmentation step, which leads to suboptimal performance. In this work, we obviated the need for large amounts of annotated data by proposing a few-shot learning process for U-Net that included a dynamic re-training mechanism. The proposed approach dynamically modified U-Net parameters to fit a small number of incoming annotated samples. In addition, our learning scheme allowed the dynamic adjustment of model parameters with respect to experts’ interaction, whereas current methods do not allow fine-tuning of the model weights after its training.

## 3. Materials and Methods

The detection of COVID-19 symptoms in CT images could be classified as a binary approach: the negative class consists of regions without induced symptoms (e.g., swelling, lesions and other types described in the introductory section), while the positive class includes areas depicting the manifestation of symptoms related to COVID-19.

Such semantic segmentation problems can be implemented in a two-step approach: (a) feature extraction over image patches and (b) a training procedure using annotated datasets. In such a scenario, each pixel is described by locally extracted feature values over a typically small area denoted as a “patch”. Deep learning techniques do both steps for a given set of data. The main question, thus, involves the type of deep-learning: traditional CNNs over image patches or FCNs over the entire image [19].

In the former case, a classifier was fed these feature values and produced an outcome that classified the pixel at the center as positive or negative. As such, for any CT slice (image) of size 630 × 630 pixels, and given a patch size of 31×31 pixels, we should annotate (630−15−15)×(630−15−15)=360,000 overlapping image patches. Deep learning feature extraction has been the common case approach, and experimental results indicate its benefits over traditional, hand-made, feature extraction. In such case, a CNN classifier could annotate the image (during the testing phase) within a few seconds even in cases of complex application scenarios [42]. The advantages of such a technique are a high accuracy rate and flexibility in handling unbalanced datasets.

The latter case involves the use of the entire image and annotation in one pass. For this purpose, fully convolutional neural network techniques were considered and implemented. The main advantages of such processes are described in the next section.

**Figure 3:**
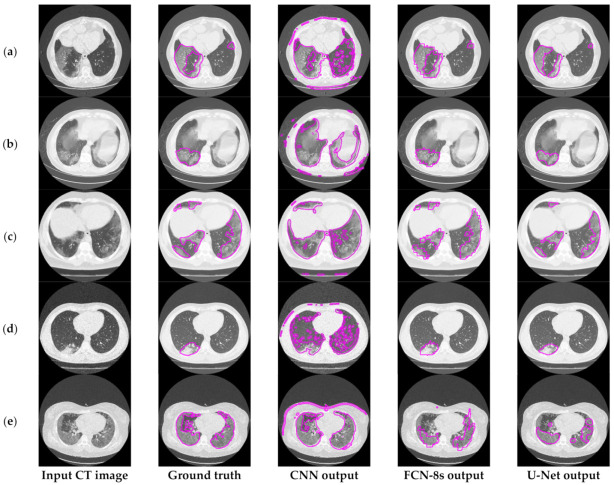
Comparison of the semantic segmentation results among deep learning models’ outputs and experts’ annotations, for different COVID-19 patients (**a**–**e**).

Figure 3 depicts an approach for the semantic segmentation of CT scans regarding the localization of the COVID-19 infected areas. In this figure, we depict the input CT signals as well as the ground-truth data. It should be mentioned that sample images of this figure correspond to different patients of the dataset. In the ground-truth column, we depict the segmentation results of CT scans referring to COVID-19 infection as performed by experts. The remaining three columns present segmentation results regarding three different methods; a CNN structure, an FCN model, and a U-Net. The CNN performs local data processing (see below) and, therefore, depicts normal lung issues as infected, one of which ruined the overall segmentation accuracy. On the other hand, the FCN and U-Net models performed better compared to the CNN structures. This is mainly because they have multiscale capabilities and are therefore able to process global and local information simultaneously. Between the FCN and U-Net models, the U-Net is a better classifier for medical imaging since it provides a more efficient representation of the local information. However, as depicted in Figure 3, there are some misclassified segmentation results where the U-Net model failed to accurately extract the COVID-19 infected areas (see the last row of Figure 3). For this reason, a new framework is proposed in this paper, called the “few-shot U-Net” model, which refines the initially trained parameters of the U-Net to increase its performance in regions where the initial U-Net structure failed. More specifically, few-shot data samples were provided by the user to retrain the network structure in cases where the performance was not so accurate.

## 4. Employed Deep Learning Techniques: Moving from Local Processing to Global-Local Analysis

There are various levels of granularity in image understanding, starting from a coarse-grained down to a more fine-grained comprehension. The first step is classification. In this case, we just indicated if an image depicted a COVID infection. The second step included localization, where along with the discrete label (COVID-19/not COVID-19), we expected a bounding box indicating the area of interest. That way, the model assisted the experts by narrowing the time they had to spend on scans.

However, for many applications (e.g., precise tumor detection) bounding boxes do not suffice. In such cases, we need highly detailed, pixel-level information, the so-called “pixel-based segmentation”. This is the goal of semantic image segmentation algorithms. In this case, we labelled each pixel of an image with a corresponding class of what was being represented. Semantic segmentation comes with specific limitations in the form of time constraints, limited hardware access, and low false-negative detection thresholds.

### Local vs. Global-Local Processing

**Figure 4:**
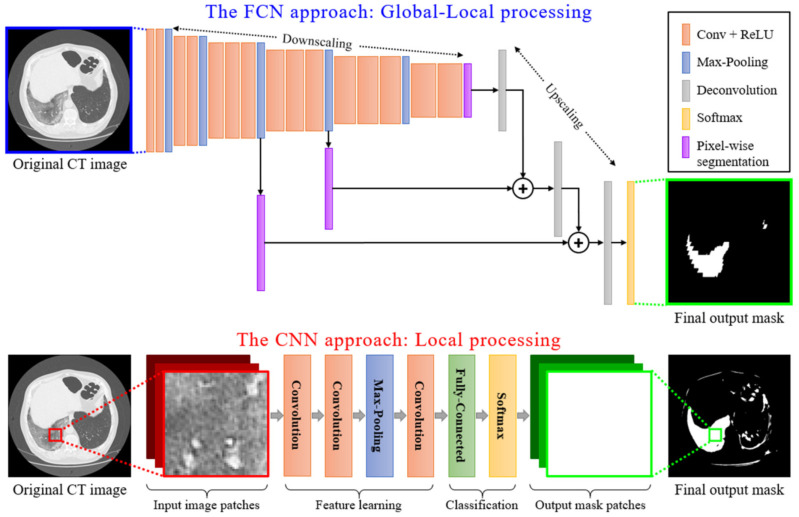
A schematic representation of local processing performed by a CNN model versus the global-local analysis performed by an FCN model.

Local Data Processing: Among the numerous deep learning techniques, Convolutional Neural Networks (CNNs) are actively applied for medical imaging problems. These include tasks related to semantic segmentation, computer-aided diagnosis, disease detection, and classification. The traditional CNN models (see Figure 4) present local processing of the image regions. In particular, the input of the CNN detector consisted of overlapping window patches (e.g., 31×31 pixels). Then, the model produced an output that classified the central pixel of each patch as positive or negative detection based on the contribution of all neighboring pixels within the patch. However, this type of local processing is not adequate for efficient semantic segmentation of medical imaging since the global image characteristics are lost.

Global-Local Data Processing: In contrast, an FCN model initially performs a multi-scale image processing through which feature maps are extracted at multiple scales. An FCN model, as the name suggests, is built using locally connected layers, such as convolution, pooling, and upsampling [43]. This is depicted in Figure 4, where a conventional CNN structure type of processing is compared to an FCN type of processing. The topology of an FCN contains 2 parts: (a) a downsampling path, which is responsible for capturing semantic/contextual information, and (b) an upsampling path, responsible for recovering spatial information. Note that no dense layer is used in this kind of architecture, which reduces the number of parameters and computation time [44,45]. Any disadvantages related to information loss due to pooling or downsampling layers can be mitigated using an operation called skip connection, which bypasses at least one layer. This type of structure enforces an FCN model to operate under a global-local data processing framework. It is clear that global-local analysis is a better classification framework for COVID-19 CT image segmentation, compared to a simple local-based one, as CNN does. Similar structures with FCNs are U-Nets, which preserve local-data features better since their connections are designed in a way to retain local information in the upsampling process [24]. For this reason, in this paper, we adopted a U-Net model as the basic structure for the classifier performing the COVID-19 segmentation of CT scans.

## 5. U-Nets for COVID-19 Segmentation

The U-Net, a variation of a CNN, was designed and applied in 2015 to process biomedical images [24,46]. Whereas a general convolutional neural network focuses its task on image classification, where input is an image and output is a label, in biomedical cases, it is required to determine not only if there is a disease but also to localize the area of abnormality, thereby performing global-local processing instead of local processing.

The U-Net is built upon the Fully Convolutional Network and modified so that it yields better segmentation in medical imaging. To that extent, the architecture contains two paths. The first is the contraction path (also known as the encoder), which is used to capture the context in the image. The encoder is just a traditional stack of convolutional and max-pooling layers. The second is the symmetric expanding path (also known as the decoder), which is used to enable precise localization using transposed convolutions. Contracting and expanding paths are connected using a bottleneck built from 2 convolutional layers (with batch normalization), with dropout.

**Figure 5:**
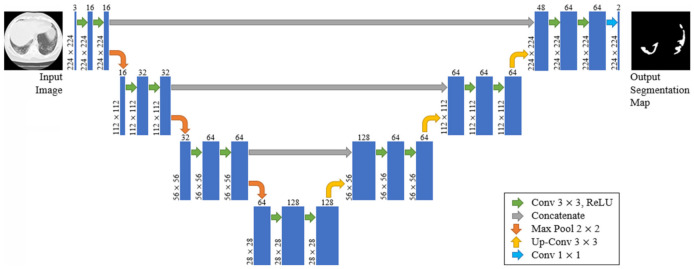
The proposed U-Net architecture.

Compared to known FCN approaches (e.g., FCN-8s) [43], the two main differences are symmetry and connection-skipping between paths. More specifically, the U-Net is symmetric (see Figure 5), and the skip connections between the downsampling path and the upsampling path apply a concatenation operator instead of a sum. These skip connections are meant to provide local information to the global information while upsampling. Given the model’s symmetry, the network has a large number of feature maps in the upsampling path, which allows transferring information. Therefore, U-Nets are structures with better capabilities for retaining local information and fusing it with the global one.

**Figure 6:**
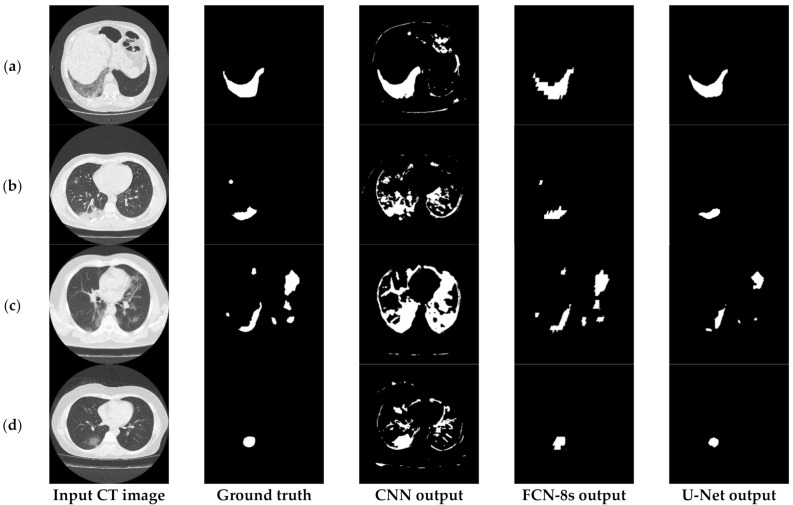
Visual comparison of the deep models’ outputs, for different COVID-19 patients (**a**–**d**). The leftmost column is the original CT scan image, whereas the second column illustrates the corresponding segmentation for COVID-19 symptomatic areas. The last two columns depict the generated semantic segmented area.

Figure 6 provides further insights into the accuracy and edge smoothness of the models’ annotations. Given a CT scan slice, the FCN-8s model tended to produce coarser boundaries. On the other hand, the U-Net provided smoother regions that were slightly smaller than the original annotated area. Both models were capable of localizing well for the majority of symptomatic regions. In contrast to the two aforementioned techniques, as shown in the third column of Figure 3 and Figure 6, the CNN model tended to produce many false positives. This means that the CNN annotated many regions as symptomatic, but most of its predicted outcomes were incorrect. It is noted that this flaw appeared mainly due to the local processing characteristics of the CNN. Thus, the predictive power of the specific deep network diminished significantly since it was most likely to cause false detections, which are critical for medical imaging applications such as the segmentation task of COVID-19 lesions in chest CT images.

### The Proposed Few-Shot U-Net Model

The main limitation of the aforementioned traditional deep models in the COVID-19 segmentation task is the assumption of stationarity in the training input samples. In particular, a set of ground-truth data was used for training the network and thus computing its respective parameters. Then, the parameters of the network were assumed to be fixed and its performance was evaluated over the data of the test set. However, such a static learning approach was not capable of improving its classification accuracy to data following a different statistical distribution compared to the learning approach of the training set. For this reason, semi-supervised learning approaches have been proposed for object detection and classification [47]. Few-shot is an alternative to the semi-supervised learning paradigm, which has recently been introduced into machine learning and computer vision research.

**Figure 7:**
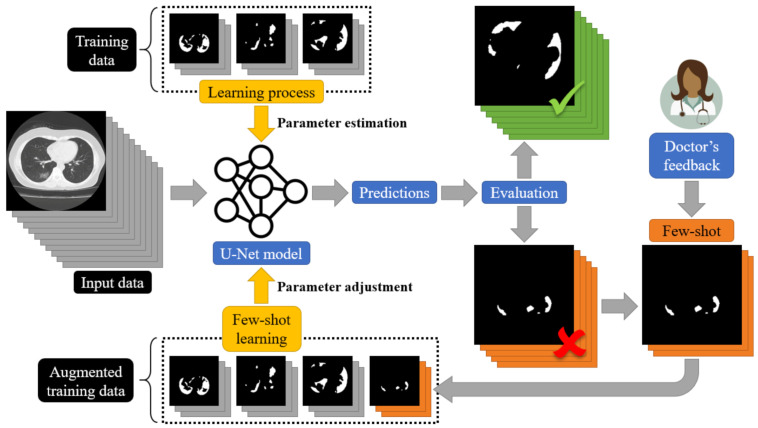
A schematic representation of the proposed few-shot U-Net for the segmentation of COVID-19 infected areas.

To address this limitation and to design a dynamic training framework for COVID-19 lung segmentation, we propose a few-shot learning procedure for U-Net networks (see Figure 7). More specifically, end-users who are medical professionals selected a few misclassified data samples, and then a retraining mechanism was carried out. This way, the parameters of the U-Net structure were dynamically modified to fit the current incoming samples, while a minimum degradation of the previous network knowledge was encountered.

The proposed architecture of the few-shot U-Net model is depicted in Figure 7. In particular, we assumed that a training set $S_{init}\;$ = $\;\left\{\left(x_{i}\left(n\right),t_{i}\left(n\right)\right)\right\}$ included pairs of input-target relationships at a time instance n. Variable $x_{i}\left(n\right)$. expresses labelled input data (CT scans of lung regions), while targets $t_{i}$ are supervised (desired) outputs (COVID-19 segmentation of the lung CT scans), provided by the medical experts. Using this initial training set, a set of parameters for the U-Net structure was extracted through the application of a conventional learning paradigm. Then, the segmentation performance of the U-Net was evaluated over a test set that included data samples outside of the ones that the network had been trained with. The performance of the trained U-Net model was evaluated by experts (medical professionals). Segmentation results of low accuracy were evaluated by the experts and corrected, and then a new augmented training set was created: $S_{Update}\;$ = $\;\left\{\left(x_{new}\left(n\right),t_{new}\left(n\right)\right)\right\}\cup^{\;}S_{init}$. The new augmented training set was used to train the network further. We assumed that a few samples were selected by the experts at a time. The network started its training from the point where the learning algorithm had been stopped and a few training epochs were considered. In this way, the proposed **few-shot U-Net** network is trained in a way to trust the new incoming (few) samples, while simultaneously a minimum degradation of the previous network knowledge is encountered.

## 6. Experimental Results

All networks were developed in Python using TensorFlow and Keras libraries. The models were trained using an NVIDIA Tesla P4 GPU provided by Google Colab. For the evaluation process, we conducted tests on a typical PC with 8 CPU cores (AMD FX-8320 @ 3.5 GHz) and 8GB RAM. Figure 4 describes the adopted topologies for the FCN-8s and the proposed CNN classifier, whereas Figure 5 presents the architecture of the U-Net model. Additionally, Figure 7 depicts the architecture of the new proposed few-shot U-Net with the main purpose of improving the accuracy of the traditional U-Net networks in the case of COVID-19 CT images. It is noted that the final few-shot U-Net model requires less than 8 MB of storage space.

### 6.1. Dataset Description

The dataset used in this work was collected from Radiopaedia [48] and manually annotated in the works of [49,50]. All images of the dataset are lung CT scans, with a dimension of 630 × 630 pixels. The data and images were labelled, segmented, and verified by radiologist medical experts. In particular, the infection was first delineated by junior annotators with 1 to 5 years of experience then refined by two radiologists with 5 to 10 years of experience. Finally, all annotations were verified and refined by a senior radiologist with more than 10 years of experience in chest radiology [50]. The dataset consisted of 939 cross-sectional images. From these, 492 slices were labelled as positive and 447 as negative. It should be noted that in positive CT images, the proportion of infections in the lungs ranges from 0.01 to 59% [50]. From the whole number of images in the dataset, 85% were used for training the deep networks, and the remaining 15% were used for testing. Among the training data, 90% of them were used for training, while the remaining 10% were used for validation. To avoid training bias and guarantee replicability of the results to other datasets, it should be noted that we applied a 4-fold cross-validation scheme within the training set. Typically, as is mentioned in [51,52], a 4- or 5-fold cross-validation is often used for segmenting lung images of COVID-19 data. It was also noted that the male-to-female ratio was 5:3. The median age was 50 years within a range of 25 to 80 years. Their clinical presentations included fever (87.5%), dyspnea (75%), cough (62.5%), chest pain (12.5%), and fatigue (12.5%).

### 6.2. Implementation and Limitations of Mitigation Strategies

Prior to any implementation approach, we should consider the limitations of the problem at hand. In deep learning approaches, there are two main concerns: data availability and data imbalance, both of which affect classification model selection and topology complexity. Although the positive-to-negative ratio of the CT samples was balanced (492:447), the total area (number of pixels) of COVID-19 versus non-COVID-19 was not. This was because in most images only a small percentage of the lung area was infected by COVID-19 (see Figure 3 and Figure 6). Therefore, the initial step was a balancing strategy for the training data, which involved undersampling of the majority class (non-COVID-19 areas) [53]. In particular, the 447 images that contained only negative annotated pixels were excluded from the training process. The remaining 492 images, which had ratios ranging from 0.01 to 59% of positive-annotated to total-image pixels, were used for training the deep networks.

Human-made annotations tended to be prone to errors [54]. Being able to distinguish in which of the two classes (positive or negative) a specific pixel belonged, was arduous, especially if the specific pixel were located on a boundary area. To do so, we used the networks’ capabilities to generalize and handle the noise, given that the erroneous annotations tended to be limited. Alternative approaches that were considered were implementing various performance metrics during the training procedure and building networks of limited complexity.

The hyperparameters of the proposed deep learning models were optimized and tuned using the Bayesian approach of [55] to maximize their classification accuracy. An alternative approach was to apply the method of [56]. It should be mentioned that in the following experiments, no initial pre-processing procedure of the image samples was considered since this was performed by the convolutional kernels of the networks. Recently, however, fuzzy logic methods have been introduced as a pre-processing scheme, such as image enhancement, to improve classification performance [57,58]. In light of recent events concerning the pandemic, it was noted that such methods have also been investigated for COVID-19 classification [59].

### 6.3. Experiments and Comparisons

#### 6.3.1. Comparisons with Other Deep Learning Models

**Figure 8:**
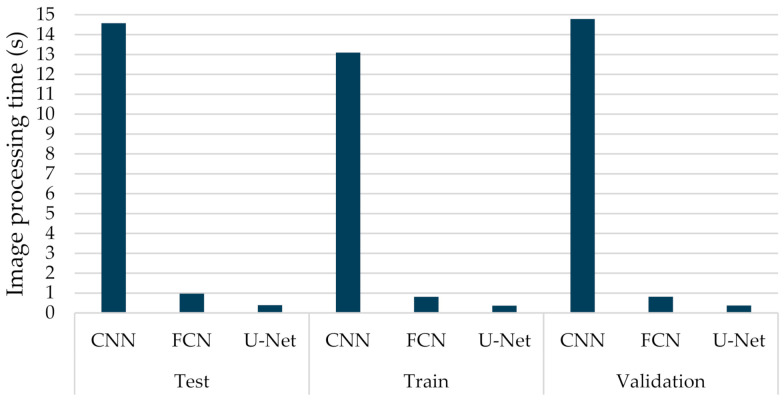
Average computational processing of different types of deep learning networks (CNN, FCN, U-Net) for COVID-19 segmentation.

By employing different classification-related performance metrics, the experimental results considered both the computational average time required by a trained network to fully annotate a CT image, and the identification capabilities. Figure 8 provides the average execution time per sample, which ranged from 0.364 to 0.970 s, for the FCN-8s and U-Net models. As was observed, the U-Net structure presented the lowest computational execution compared to the other compared models. More specifically, the CNN model was the most time-consuming, having computational times per image that exceed 13 s. The computational complexity of the retraining task of the U-Net model is pretty low, which is also computationally efficient. This is mainly due to the fact that only a few of the new incoming data were considered in the retraining process. Therefore, the proposed few-shot U-Net framework retained the computationally efficient characteristics of the original U-Net model.

**Figure 9:**
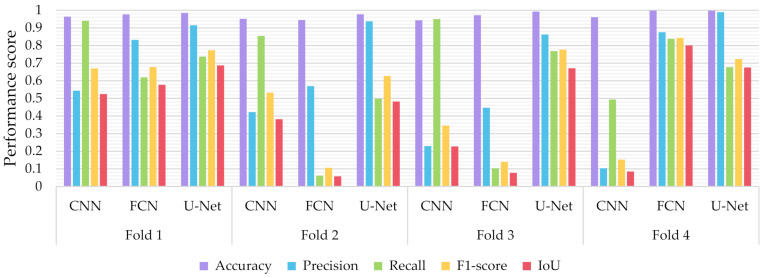
Performance metrics for accuracy, precision, recall, F1-score, and IoU for the different semantic segmentation approaches in the test set.

**Figure 10:**
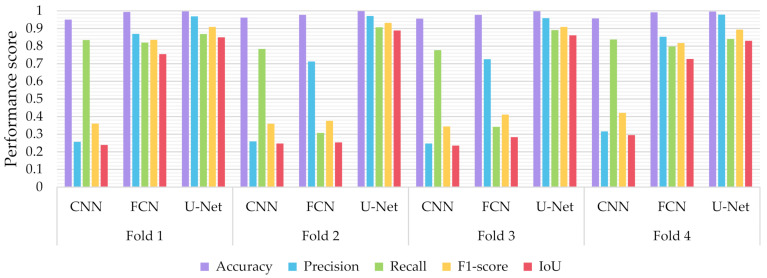
Performance metrics for accuracy, precision, recall, F1-score, and IoU for the different semantic segmentation approaches in the training set.

**Figure 11:**
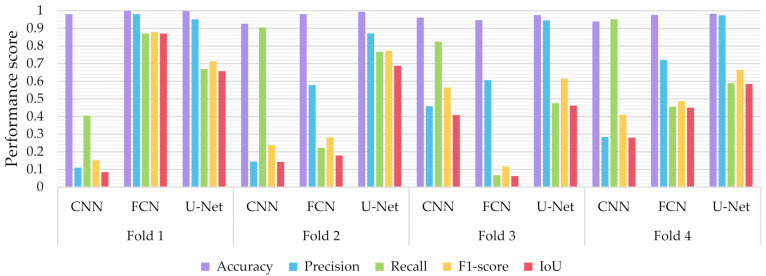
Performance metrics for accuracy, precision, recall, F1-score, and IoU, for the different semantic segmentation approaches in the validation set.

Similar to the evaluation strategies adopted in other classification related problems, five performance metrics were considered: (a) accuracy, the percentage of correct classification for both positive and negative classes; (b) precision, the number of correct positive predictions; (c) recall, the fraction of positive samples that were successfully retrieved; (d) F1-score, the weighted harmonic mean of precision and recall; and (e) IoU, the ratio of the intersection to the union of the predicted segmentation mask and the ground truth. In Figure 9, Figure 10 and Figure 11 we depicted the performance scores for these metrics for the three compared deep learning models: the CNN, the FCN, and the U-Net. In the aforementioned figures, we illustrated the results within the training, validation, and test sets respectively, for each of the four folds of the cross-validation.

As observed in Figure 9, Figure 10 and Figure 11, the U-Net model presented better segmentation accuracy for the COVID-19 infected areas compared to the other deep learning models. It was noted that the difference between accuracy and F1-score as well as IoU can be put down to class imbalance. Indeed, the majority class (no detections) was almost always correctly identified. The false-negative detections could be spotted on the boundaries of the infected areas in images where COVID-19 symptoms were clearly manifested. Yet, the F1-score and the IoU were relatively high, thus indicating that the minority class (COVID-19 symptomatic areas) could be identified.

Recall indicates the network’s capability to identify the case; that is, if a CT image has COVID-19 infected areas, the model will indicate these areas even if precision (on the positive class) is limited. Our results (Figure 9, Figure 10 and Figure 11) indicated two important features: (a) significant recall variation scores among train, validation, and test sets and (b) better generalization capabilities of the U-Net model compared to the CNN and FCN-8s.

Since false detections are critical in medical imaging applications, the present paper aimed to minimize false positives (normal areas diagnosed as symptomatic). Moreover, due to the pandemic, there is a growing need for chest CT scan interpretation. In this context, we focused on reducing the workload of radiologists by aiming for a high percentage of true positives (symptomatic areas diagnosed as symptomatic). Within the framework of these criteria, we had to exploit techniques that were able to achieve very high precision scores and satisfactory recall scores. Although all three models provided similar results for IoU, F1-score and accuracy in some cases, the FCN-8s and U-Net models outperformed the traditional CNN, due to their high-precision scores, which involved both FP and TP values.

**Figure 12:**
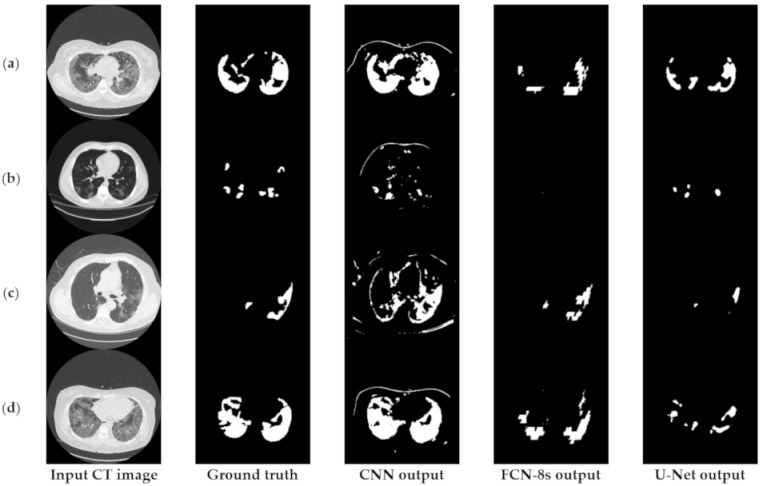
Relatively low detection performance on challenging images. More specifically, FCN-8s and U-Net show low detection accuracy in (**a**,**b**) and (**c**,**d**) respectively.

Figure 12 presents the segmentation results of the COVID-19 infected areas for the three deep learning models. We observed that the CNN results presented over-segmentation, including also regions which were not infected by COVID-19. Additionally, the FCN and U-Net showed improved segmentation performance, with U-Net providing the best segmentation results. In this figure, we provided an indication of low segmentation performance. Detection failures may have included partial area annotation or no annotation at all, in spite of the appearance of symptoms in the CT image. Nevertheless, axial CT scans consisted of consecutive slices, so even if the identification failed for a given image, it was highly likely that it would have succeeded in neighboring ones. In order to address these limitations and provide a potentially valuable aid for medical professionals, we proposed the so-called few-shot U-Net model, the performance of which is presented in the next subsection.

#### 6.3.2. The Performance of the Proposed Few-Shot U-Net Model

For the few-shot learning, we selected 8 images of the test set (5% of the total test images), in which the deep network showed the worst results for F1-score and IoU. These images were extracted from the test data and added to the training set, thus creating a slightly extended training dataset. The U-Net model was retrained for only 5 epochs during the rectification process to prevent overfitting on the extended training data. It should also be noted that we reduced the initial learning rate by a factor of 10 to avoid damaging the reused weights of the initial network.

**Figure 13:**
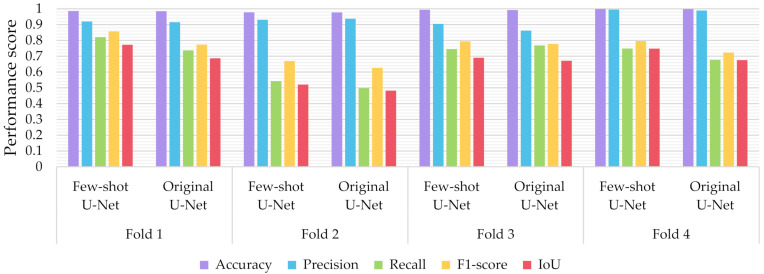
Performance metrics for accuracy, precision, recall, F1-score, and IoU of the proposed few-shot U-Net model against the traditional U-Net over the test set.

Figure 13 depicts the performance scores of the U-Net model before and after rectification. In particular, the rectified U-Net showed improved performance on the test set (now consisting of the original test data minus the 8 extracted images), in recall score (increment of 4.409 ± 4.790%), while preserving high precision (change by 1.162 ± 2.137%), resulting in an overall F1-score increment of 5.394 ± 3.015% over the 4-fold cross-validation. Similarly, the proposed few-shot U-Net presented an IoU improvement of 5.388 ± 3.046% for all test data. Similarly, it was noted that the deep network maintained its high-performance metrics on the train and validation datasets, thus avoiding overfitting on the extended training data.

**Figure 14:**
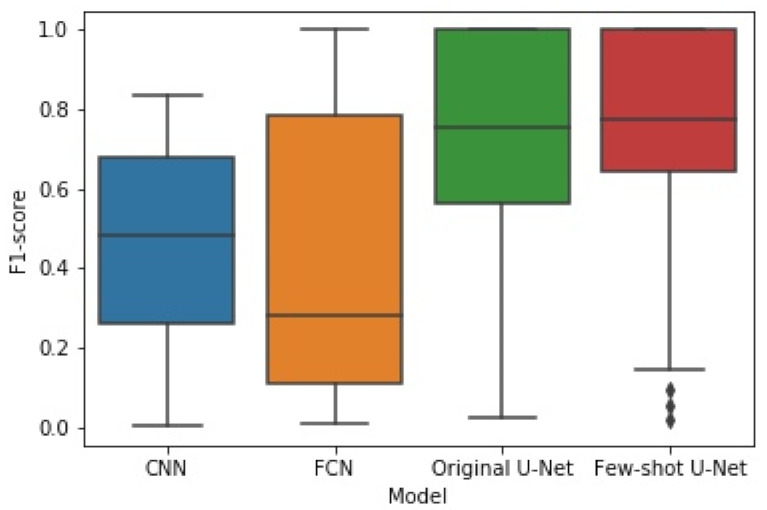
The box plot of experimental results for F1-score obtained by the four methods.

**Figure 15:**
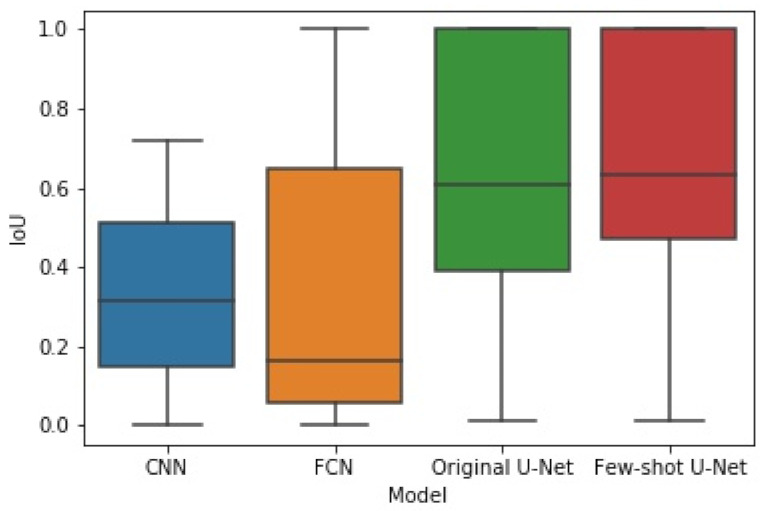
The box plot of experimental results for IoU obtained by the four methods.

The box plots of Figure 14 and Figure 15 illustrate the distribution of the F1-score and IoU, respectively, for each of the employed networks by using results from the different cross-validation folds. It can be seen that the distribution of both F1-score and IoU of the proposed few-shot U-Net was superior to those of the traditional static CNN, FCN, and U-Net models.

To examine whether the improvement of the few-shot U-Net performance over the conventional U-Net was statistically significant, we used the Kruskal-Wallis test on the obtained F1-score and IoU results from different cross-validation folds. Kruskal-Wallis is a rank-based nonparametric test that can be used to determine if there are statistically significant differences between two or more groups of an independent variable on a continuous or ordinal dependent variable [60]. This method is considered to be a nonparametric alternative to the one-way ANOVA and an extension of the Mann-Whitney U test to allow the comparison of more than two independent groups [61]. We selected the Kruskal-Wallis test since it is designed to compare multiple groups that are unpaired and not normally distributed. In particular, as illustrated in Figure 14 and Figure 15, the F1 and IoU scores of the test data do not follow a normal distribution. Since the *p*-value of the Kruskal-Wallis test was 0.026 (less than 0.05), we concluded (with a confidence level of 95%) that there was a significant difference in the comparative results of the few-shot and traditional U-Net models.

**Figure 16:**
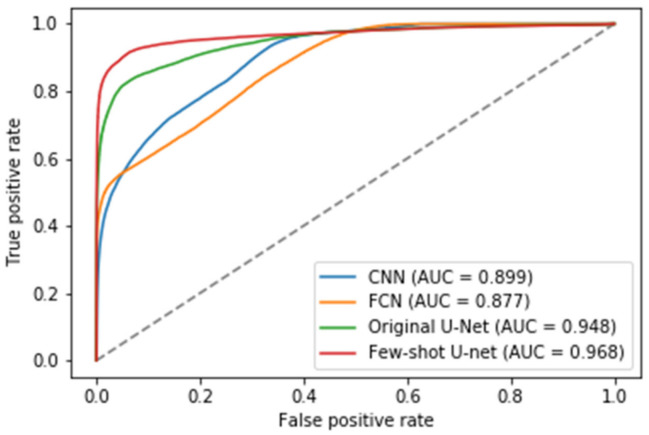
The Receiver Operating Characteristic (ROC) curves for the traditional deep learning models (U-Net, FCN and CNN) and the proposed few-shot U-Net along with the corresponding Area Under Curve (AUC) scores.

In addition to the metrics comparisons, we presented the Receiver Operating Characteristic (ROC) curves of the different methods to illustrate their diagnostic ability as their discrimination threshold was modified (see Figure 16) [62]. To evaluate the quality of the prediction models we also reported the AUC (Area Under Curve) values for the test data. The higher the AUC score, the better a classifier performs in a pixel-wise classification task. More specifically, an AUC of 0.5 corresponds to a deep model that produces random classification results, while an AUC of 1 denotes a perfect classifier. Figure 16 shows the superiority of the few-shot U-Net architecture AUC values over those of the traditional static U-Net, FCN, and CNN models.

**Figure 17:**
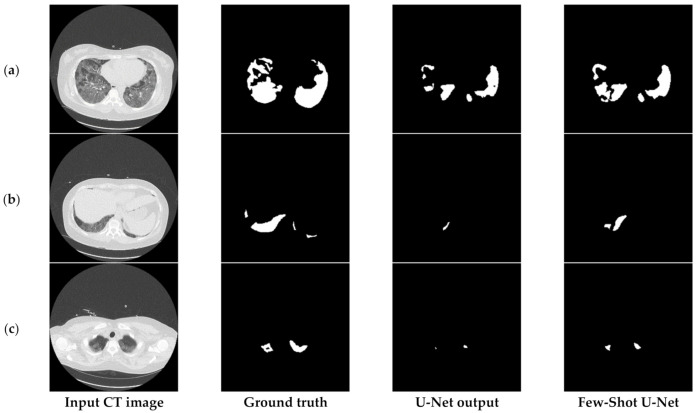
Visual comparison of the output masks produced by the proposed few-shot U-Net model against the traditional U-Net, for different COVID-19 patients (**a**–**c**).

Finally, Figure 17 compares the output of the U-Net model on challenging images before and after applying the rectification mechanism. More specifically, in such cases the initial network managed to identify the infection but at the same time failed to determine the precise shape of the consolidation (third column of Figure 17). However, through the rectification process and the weight adaptation of the U-Net model, the reduction of false negatives was achieved (last column of Figure 17), thus providing a more concrete and effective solution to the COVID-19 segmentation problem. Therefore, the few-shot U-Net model improved the segmentation performance over those CT scans. Moreover, it provided a dynamic training mechanism for the traditional static U-Net model. This means that new incoming few samples can be used to re-configure the model to fit dynamic changes of the environment.

## 7. Discussion

In this paper, we proposed a few-shot U-Net deep learning model for detecting infectious COVID-19 regions in CT scans. The traditional approaches for COVID-19 CT scan segmentation used a supervised learning paradigm. However, such approaches require, large volumes of data for training, and they assume fixed network weights for the testing phase after the completion of the model training. Collecting large amounts of training data is an arduous task, especially in the medical imaging domain, since it requires manual annotation. To overcome the aforementioned difficulties, the concept of few-shot learning has recently been adopted to train a model based on a few, instead of a large number of, training samples. Few-shot learning involves a general pool of algorithms with many variations depending on the problem, the degree of user interaction, the complexity of the data and so on.

In this context, we combined the idea of few-shot learning with a U-Net model for COVID-19 CT scans segmentation. We introduced a novel algorithm for implementing few-shot learning in which the network weights (parameters) were dynamically adjusted as new incoming few samples were fed into the network. Therefore, it is an on-line learning paradigm, exploiting few samples at a time for training. The new incoming inputs stem from interaction with an expert who evaluates the network results during the testing phase and points out the misclassified outcomes.

Although our methodology improved segmentation performance and introduced an on-line learning paradigm, it required expert feedback (interaction) during the testing phase of the algorithm; that is, the new incoming inputs that were used to adjust the network weights came from medical experts who evaluated the network results during the testing phase pointing out the misclassified outcomes. Therefore, our method assumed that humans are in the loop of the on-line learning phase. Mistakes in the decisions of the medical experts, though not probable, would have led to a deterioration in the overall network performance. The same problem was also encountered in the supervised learning paradigm in the case of erroneously annotated data. However, the ground-truth data in supervised learning were constructed off-line; therefore, there was plenty of time to re-evaluate the annotation accuracy. Instead, in the proposed learning scheme, the dynamic weight adjustment was carried out on-line, so there is no time to reassess the expert interactive decision. Another limitation of our scheme was that as new training samples came in, forgetting mechanisms were required to reduce the size of the training dataset.

## 8. Conclusions and Future Work

In this paper, we presented a few-shot learning paradigm for segmenting COVID-19 infectious regions. Our model was based on a U-Net structure, which was innovatively modified to allow a few new incoming samples to re-configure the network parameters. This retraining procedure allowed the model to trust as much as possible the new incoming data, while simultaneously minimally degrading the existing knowledge. The main difference of the proposed algorithm compared with the traditional approaches is that it is an on-line learning paradigm, not the static supervised learning of U-Net. We call this novel approach “few-shot driven U-Net” and it seems to be efficient and effective at segmenting infectious COVID-19 areas.

Experimental results indicated the effectiveness of the proposed few-shot learning combined with a U-Net model for detecting and segmenting infectious COVID-19 regions. Comparisons with other deep learning models, such as Fully Convolutional Networks (FCNs), Convolutional Neural Networks (CNNs) and the traditional U-Net structure without the use of a few-shot learning paradigm, showed that the proposed few-shot driven U-Net is a promising Artificial Intelligence (AI) paradigm for medical imaging, especially for detecting infectious COVID-19 regions.

Based on the experimental results, our main conclusions are the following:

1. The proposed few-shot U-Net model, using 4-fold cross-validation results of the different classifiers, presented an IoU increment of 5.388 ± 3.046% for all test data compared to that of a conventional U-Net.
2. Similarly, regarding the F1-Score, we observed an improvement of 5.394 ± 3.015%. As far as the precision and recall values were concerned, we observed an increment of 1.162 ± 2.137% and 4.409 ± 4.790% respectively.
3. The *p*-value of the Kruskal-Wallis test on the obtained F1-score and IoU results, was 0.026 (less than 0.05) between the proposed few-shot U-Net model and the traditional one. That implies, with a confidence level of 95%, that a significant difference exists in the metrics of the two methods.
4. The proposed model required few new incoming samples and roughly 8 images to efficiently adapt its behavior.
5. The computational complexity of the proposed few-shot U-Net model was similar to that of the traditional U-Net since the new incoming data were combined with the previous samples to improve the generalization capabilities of the network.

Of particular interest for future work is the interweaving of few-shot learning with other deep models and learning schemes, such as the concept of Transformers [63]. Recent studies have concluded that transformer-based models perform better than other types of networks, like recurrent and convolutional structures, in a variety of visual data benchmarks.

## Figures and Tables

**Table 1:** Summary of machine learning techniques employed for COVID-19 detection/segmentation in CT scans.

Techniques/Models	Works	Number of Classes
Convolutional Neural Networks (CNN)	[14]	2 (COVID-19, non-pneumonia)
	[20]	3 (COVID-19, CAP, non-pneumonia)
	[32]	2 (COVID-19, SARS)
U-Net	[6,25,28]	2 (COVID-19, non-pneumonia)
LSTM-CNN	[22]	2 (COVID-19, non-pneumonia)
CNN + Fuzzy Inference System	[31]	2 (COVID-19, non-pneumonia)
ResNet50	[26]	3 (COVID-19, CAP, non-pneumonia)
AlexNet, Inception-V4	[30]	2 (COVID-19, other disease)
AlexNet, VGG-16, VGG-19, SqueezeNet, GoogleNet, MobileNet-V2, ResNet-18, ResNet-50, ResNet-101, and Xception	[29]	2 (COVID-19, non-pneumonia)
Volumetric Medical Image segmentation networks (V-Net)	[37,40]	2 (COVID-19, non-pneumonia)
Random Forests	[41]	3 (COVID-19, CAP, non-pneumonia)
Genetic Algorithm + Naïve Bayes	[35]	2 (COVID-19, non-pneumonia)
Type 2 fuzzy clustering + Fuzzy Modified Flower Pollination Algorithm	[36]	2 (COVID-19, non-pneumonia)

## Data Availability

The CT scans are public, collected by Radiopaedia (link: https://radiopaedia.org/, accessed date: 12 February 2021). The aforementioned data used in our study were also manually annotated in the work of Jun Ma et al. “Towards Data-Efficient Learning: A Benchmark for COVID-19 CT Lung and Infection Segmentation”. The annotated dataset was obtained by the open-access repository Zenodo, developed under the European OpenAIRE program and operated by CERN (link: https://zenodo.org/record/3757476#.YC47I2j7SUm, accessed date: 12 February 2021).
